# Diabetes self-management among Arab Americans: patient and provider perspectives

**DOI:** 10.1186/s12914-016-0097-8

**Published:** 2016-08-31

**Authors:** Heather Fritz, Rosanne DiZazzo-Miller, Elizabeth A. Bertran, Fredrick D. Pociask, Sandra Tarakji, Judith Arnetz, Catherine L. Lysack, Linda A. Jaber

**Affiliations:** 1 Institute of Gerontology, Wayne State University, 87 East Ferry Street, 226 Knapp Building, Detroit, MI 48202 USA; 2 Department of Health Care Sciences, Wayne State University, 259 Mack Ave, Detroit, MI 48201 USA; 3 Department of Pharamacy Practice, Wayne State University, 259 Mack Ave, Detroit, MI 48201 USA; 4 Department of Family Medicine and Public Health Sciences, Wayne State University, Detroit, MI 48202 USA; 5 Department of Family Medicine, Michigan State University, 788 Service Rd., B103 Clinical Center, East Lansing, MI 48824 USA

**Keywords:** Diabetes mellitus, Vulnerable populations, Focus groups, Primary health care, Minority groups

## Abstract

**Background:**

Arab Americans have a high burden of diabetes and poor outcomes compared to the general U.S. population. Diabetes self-management (DSM) requires a partnership between patients and providers that fosters mutual understanding and shared decision-making. Cultural factors influence this process; however, little is known regarding the cultural impact on DSM or if perceptions differ between patients and providers.

**Methods:**

Qualitative content analysis was used to analyze five focus groups–two groups with Arab American providers (*n* = 8) and three groups with adult Arab Americans with diabetes (*n* = 23). Focus groups examined patient and provider perspectives on the meaning of DSM and cultural barriers and facilitators among Arab American patients.

**Results:**

Four distinct themes included limited resources for DSM education and support, stigma as a barrier to ongoing support, family support as an opportunity and challenge, and Arab American patient-provider relationships.

**Conclusions:**

Findings indicate several domains should be considered for clinical practice including a need to develop linguistically and culturally reliant educational materials and relevant supports for use in the Arab American population. Findings highlight differing views among providers and patients on the familial role in supporting DSM efforts and why some patients feel dissatisfied with clinical encounters.

## Background

Approximately 90–95 % of the everyday management of diabetes is under the control of the patient [1]. Nonetheless, providers play a primary role in fostering DSM vis-à-vis partnering with patients to develop reasonable DSM approaches and recommending relevant programs and services [2, 3]. Optimizing diabetes outcomes, therefore, entails providers and patients working together on DSM from a position of mutual understanding and shared decision-making. Culture inherently influences these processes and subsequently, the ability to work in partnership toward optimal diabetes-related goals. Moreover, the impact of culture may be especially significant among populations such as Arab Americans, who hold unique health beliefs and behaviors that differ significantly from the cultural norms of the general US population [4–6].

The Arab American Institute [7] estimates that there are over 3.5 million Arab Americans nationwide. While underrepresented in diabetes research, the nine studies that have examined the prevalence of diabetes among Arab Americans have yielded diabetes estimates ranging from 4.8 to 33.0 % [8–16]. In Dearborn Michigan, which has the second largest population of Arab Americans in the US, one study estimated the prevalence of diabetes in the Arab American population to be 18 % [13]. In addition, another study conducted among the Dearborn population suggests that less than one third of Arab American patients with diabetes achieve targeted glycemic control, and most receive less aggressive pharmacotherapy compared to national standards [5].

Despite the impact of culture on DSM and the high prevalence of diabetes among this group, there is limited literature on these topics. Information on cultural impacts of DSM including how Arab American patients understand DSM compared to providers serving Arab American patients is critical for improving outcomes [17, 18]. To this end, we present findings from a qualitative study examining Arab American provider and patients’ perspectives of the meaning of DSM and perceived culture related barriers and facilitators to DSM and discuss how insights gained from the study could inform clinical practice and future research.

## Methods

### Design

We conducted five focus groups; two sessions were conducted with (*n* = 8) Arab American healthcare practitioners and three sessions were conducted with (*n* = 23) Arab American patients with type 2 diabetes mellitus. The focus group approach was chosen because it is an effective method to understand individual experiences and to identify factors that influence behaviors (19). Data was analyzed using an inductive approach to qualitative content analysis without a priori categories [19, 20]. The study protocol was approved by the Wayne State University Institutional Review Board.

### Setting and study participants

A convenience sample of Arab American healthcare providers, including pharmacists and physicians, were recruited from the community-at-large and from health care facilities located in Dearborn, Michigan in neighborhoods mainly populated by Arab Americans. Providers were eligible if they self-reported that they practiced in Dearborn, MI, and that ≥50 % of their patients were Arab Americans. The PI (LJ) contacted providers who practiced in the study area and if they met the screening criteria, they were invited to participate in the study. Fifteen providers were invited to participate in the study (*n* = 7 declined). We chose to focus our recruitment on pharmacists and physicians because our previous research and clinical experience suggested that Arab Americans most commonly seek diabetes-related care from these two professions.

Recruitment began by contacting Arab American pharmacists practicing in the study area. Pharmacists were provided with inclusion and exclusion criteria for participation in the study and were asked to identify and refer eligible patients that visited their pharmacy to study staff for screening and enrollment. Patient and provider participants were also recruited directly by the PI through personal contacts in the Arab American community and through word-of-mouth–approaches that have been effective when conducting previous research in this close-knit community [15]. The physician participants for our study were all recruited directly by the PI. Bilingual recruiting personnel used Arabic or English depending on the preference of the approached patient. Patient participants were included if they self-identified as Arab or Arab American, were 18 years or older, spoke primarily Arabic, and had a self-reported diagnosis of diabetes. Pregnant individuals were excluded.

### Data collection

Two digital video recorders were used to record the focus groups. The camera configuration allowed investigators to visibly identify all individual patient and provider participant responses, diminish the possibility of data loss, and effectively address limited instances of semantic noise (e.g., participants speaking at the same time).

All patient and provider participants were checked-in (i.e., patient participant focus groups were separate from provider participant focus groups), given the opportunity to ask questions, underwent written informed consent and completed the intake form and questionnaire in a reception area prior to entering a conference room that was setup in advance of participant arrival. Patient participants were requested to complete a socio-demographic and general health questionnaire. Provider participants were asked to complete a biographical questionnaire (e.g., profession, location and years of practice and educational background). The informed consent stated that focus groups would be audio- and video-recorded and notes would be taken during the session. Participants were then seated at a conference table.

Each focus group began with a brief description of the study purpose and focus group etiquette, followed by brief introductions, and the opportunity to ask questions and receive answers. The skilled moderator, a Syrian trained physician fluent in Arabic and with extensive diabetes care experience conducted the focus groups. The provider focus groups included both pharmacists and physicians and were conducted in English, (all Arab American healthcare providers were fluent in English) while the patient sessions were conducted in Arabic. Provider and patient focus groups followed the same protocol. During each session, the moderator presented the group participants with four questions specific to their perceptions of DSM, barriers and facilitators to DSM, and perceived influences of Arab American culture on DSM. A fifth and final open-ended question offered participants the opportunity to share any pertinent information or beliefs they felt were not addressed in the previous questions.

### Data analysis

All focus group discussions were transcribed verbatim. Because most of the patient focus group participants responded in Arabic, the patient focus groups were translated from Arabic to English. Next, transcripts were reviewed and compared to video recordings by the moderator to ensure accuracy of transcription. After review, the transcripts were analyzed using an inductive qualitative content analysis approach [21]. The analysis procedures began with two researchers independently reading transcripts and assigning codes to segments of text. Initial codes were then grouped into main themes. To assess inter-coder reliability the researchers then met to review their codes and discuss their findings, which revealed a consensus level of 80 %. Disagreements about codes were discussed until agreement could be reached. After this step, a third researcher who was not involved in the coding then reviewed and confirmed the final themes and sub-themes. The consolidated criteria for reporting qualitative research (COREQ), were used when planning and executing this study. Guba and Lincoln’s [22] four criteria for judging qualitative rigor were also followed.

## Results

Arab American providers included physicians (*n* = 5, mean years of clinical practice 8.00 ± 8.89 SD) and pharmacists (*n* = 3, mean years of clinical practice 19.00 ± 11.00 SD), with the majority being male (62 %) and the remaining 38 % female. Most patient participants were female (57 %) and many originated from Lebanon (43.5 %), which is the most common Arab nationality represented in Michigan [7]. Approximately half (52 %) reported no educational degree; similar educational findings have been previously reported in Dearborn [13]. The majority of patient participants (57 %) reported that their health was fair or poor which would be expected based on inclusion criteria. Four distinct themes emerged from the discussions of providers’ and patients’ perceptions of DSM: limited resources for DSM education and support; Stigma as a barrier to ongoing support; family support as an opportunity and challenge; and Arab American patient-provider relationships. Each theme is presented in further detail below. Table 1 includes illustrative quotes related to each theme.

**Table 1:** Illustrative quotes about limited resources for DSM education and support

Theme	Limited Resources for DSM Education and Support
Illustrative Quotes	*The sad thing is, I’ve seen pamphlets and I’ve seen diets they give them [Arab American patients] at the hospital. The food on there doesn’t make sense, they would never eat it in a million years. You will never find a Hajji [an elderly woman] eating a turkey sandwich. [Group 1: Provider1]* *How many dieticians do our diabetic patients go to that speak Arab fluently?…we don’t have enough resources in the community to effectively educate our patients. [Group 1: Provider 3]* *They don’t wanna take the medication, they wanna go and get… this herb or these concoctions from the Middle East. And they bring it trying to bring their sugar down, and they give it to each other and they tell each other about it.…, maybe I can take out one of the drugs and its like, no you cannot. [Group 1: Provider 2]* *No the education classes for diabetes or any disease…it will help any people around but you know what…there is classes for American Diabetes Association … but most of them, are away from [the city]… I can’t go because they are far away. [Group 3: Patient 1]* *We are not educated about the disease we have…There should be a special committee for Arab American people that have diabetes…to give us lessons, and educate us about the disease. [Group 3: Patient 5]*

### Limited resources for DSM education and support

Providers and patients agreed that a key challenge was the significant continuum of ‘gaps’ in availability and quality of culturally appropriate DSM resources for Arab Americans with diabetes, which contributed to patients not understanding the seriousness of the disease or how to perform DSM. As one provider [Group 2: Provider 4] stated, “*In the Arabic community, … most of the time they don’t understand the seriousness of a disease.* One key limitation that providers faced was the lack of linguistically and culturally appropriate DSM educational materials. Obtaining Arabic language DSM materials could be difficult and even when patients were given Arabic language materials, providers expressed that the content was not always sensitive to Arab culture.

This gap extended to the provision of supportive health services as well. Providers discussed the need for multidisciplinary teams (e.g., physicians, pharmacists, dieticians, and diabetes educators) of Arabic-speaking healthcare providers to better facilitate DSM with their patients, especially in the context of increasingly brief clinical encounters where it was difficult to comprehensively address their patients’ DSM needs. Nonetheless, most providers lacked access to such resources and had few alternatives to assist their Arab American patients.

Providers also discussed that they did counsel their patients about DSM and that they had to adapt their DSM counseling to the educational level of their patients while also having to address cultural beliefs that impeded DSM. Taken together, providers felt these limitations and barriers impacted their ability to deliver quality diabetes care and guide their patients in DSM.

While patients also emphasized that education was essential for DSM they did not explicitly discuss access to a multidisciplinary team, and with the exception of mentioning pharmacists, did not differentiate other healthcare practitioners in their discussions. Instead, patients emphasized that their primary barrier to DSM was the lack of formalized DSME programs and they perceived that a comprehensive program did not exist locally. Without access to educational resources, patients felt ill equipped to engage in DSM and actively advocated for themselves, expressing the need for more DSME programs tailored to Arab Americans. See Table 1 for examples of provider and patient illustrative quotes.

### Stigma as a barrier to ongoing support

Continuing support was also perceived as important for sustaining diabetes DSM across time. A cultural element that providers identified as being a barrier to ongoing DSM support was the cultural stigma associated with diabetes, or illness of any kind. Specifically, providers discussed that the stigma associated with disease could lead to patients avoiding acknowledging the disease or engaging in self-management activities. Providers also expressed that the impact of stigma on health could be significant because it could prevent patients from making health-promoting choices, (e.g., seeking mental health support), or cause patients to withdraw from meaningful social activities. Moreover, as one provider noted there were few supports available for patient feeling stigmatized because of their disease. Interestingly, patients did not directly discuss feeling stigmatized. Though patients discussed that they were not likely to disclose their condition in social situations, this was because social activities often centered on food and some they felt that it would be disrespectful to turn something down from someone in their house. See Table 2 for examples of provider and patient illustrative quotes.

**Table 2:** Illustrative quotes about stigma as a barrier

Theme	Stigma as a Barrier to Ongoing Support
Illustrative Quotes	*They all carry that stigma, that disease, or any kind of mental health, anything of that nature, would equate to weakness or breakdown, and that’s, … They don’t like people knowing that they’re ill. … So I think, there is just like a dirty stigma. [Group 1: Provider 1]* *They don’t talk about it. At least they might talk about it to their immediate family, but they don’t talk about it when there are functions and there are a lot of gatherings…or say, ‘I’m not going to have [eat] this’…because if they do then they’re bringing attention to themselves. [Group 1: Provider 3]* *I think the stigma clouds their judgment on making the right decisions for them to help them. For instance,…one of our patients, he is diabetic, he is 34 years old and he’s an amputee…Severe diabetic, and…he became noncompliant because of depression…There is absolutely no way he was gonna go to therapy. Because therapy is associated with weakness and the Arab community, especially, especially the men, do not wanna be associated with weakness. [Provider2: Group 2]* *But we see them like the diabetic patients who they think “Oh my gosh”, you know, “I can’t go to family functions all the family functions have to do with food. I can’t attend this wedding, I can’t go on vacation. I can’t do this. I have to sit and I have to pretend like ok I can’t eat”, you know and that’s a big stigma for them, but we don’t even offer that kind of support. [Group 1: Provider 3]*

### Family support as an opportunity and challenge

Providers and patients both expressed that family was very important in Arab culture. Nonetheless, they differed in their perceptions of how family impacted ongoing DSM. Providers repeatedly identified the family as an important source of DSM support and that without family supports patients were likely to have poor DSM. Moreover, as the following excerpt suggests, providers actively engaged family members in hopes of improving the chances of DSM success, giving examples of enlisting the assistance of families to “watch out” for and motivate patients.

In contrast, patients did not discuss their families as actively supporting their DSM efforts. Instead, patients expressed that daily life stress made their ongoing DSM difficult and often attributed daily life stress to their families. In addition, some patient participant’s linked stress to increases in their blood glucose levels. Thus, while family may support Arab Americans DSM efforts, they may also impede them as well. See Table 3 for examples of provider and patient illustrative quotes.

**Table 3:** Illustrative quotes about family involvement in DSM

Theme	Family Support as an Opportunity and Challenge
Illustrative Quotes	*Support from family you know might be something very important you know.… Educating the family members, trying to tell them about something like that. We have this issue going on. Everybody should be involved in this as a family. You know, the good thing about Arabs is that the family ties are pretty good so by educating the family members to help us do the job that’s needed, at least good control. If somebody is always watching what they do, telling them, ‘oh, watch out fruit at night is a bad idea. [Group2: Provider 2]* *Sometimes they tell you, ‘I want to eat healthy, but my mother puts everything on the table’. If they don’t eat, the mother gets mad. [Group 1: Provider 4]* *If they don’t allow the family members to help them, ultimately, and I hate to say this…they do fail because they become non-compliant. [Group 1: Provider 1]* *In our family we do not have diabetes. It is not genetic. However, in 2003 the war on Iraq we saw it live on TV, the bombing, and such, we felt that no one was alive from our families, everyone died. As a result, I got diabetes. My psychological state had an affect. [Group 1: Patient 3]*

### Arab American patient-provider communication and clinical relationships

Providers and patients also discussed that the nature of the patient-provider relationship impacted patients’ DSM efforts. Both groups expressed that patient’s held both positive and negative attitudes toward provider-patient interactions but differed somewhat in their views about why patients felt the way they did. Providers perceived that patients’ held positive attitudes about provider-patient interactions because healthcare providers enjoyed a position of respect within Arab culture. Because of their social status some patients would make extra efforts to be adherent to DSM regimens in order to please providers.

The majority of patients that held a positive view of their patient-provider relationship emphasized the provider as a respected authority, who was ultimately the best source of guidance on DSM related matters. Other patient participants with positive attitudes perceived the role of provider as less of an ultimate authority, and more as part of a team. In this view, patients emphasized they did not blame providers for poor DSM; both patients and providers held responsibility for successful interactions.

The primary reason that providers gave for their patients holding negative views of the patient-provider relationship was the tendency in Arab culture to associate physicians with illness which could lead to reluctance to seek care from medical professionals, dissatisfaction with care, and poor adherence to provider recommendations. Patients that held negative attitudes about provider-patient encounters, however, did not relate these attitudes to associating their provider with “illness”. Instead, patients’ negativity stemmed from their perceptions that Arab American providers were not as caring (compared to their perceptions of American doctors). Examples of ‘not caring’ included the perception that providers accepted more patients than they could provide care for, that they overly focused on prescribing medications while neglecting other areas of DSM and that they didn’t maintain expected follow-up with patients. See Table 4 for examples of provider and patient illustrative quotes.

**Table 4:** Illustrative quotes about patient-provider relationships

Theme	Arab American Patient-Provider Communication and Clinical Relationships
Illustrative Quotes	*As a doctor they want to try to please you…they’ll try and come back and show you that…they did what they were supposed to do. [Group 2: Provider 2]* *The doctor has to direct the diabetic patient…, Now the physician, why should you cooperate with him? Because if he found that stored blood sugar is high, he renews the medication or decreases it or gives you. [Group 1: Patient 4]* *The physician and I are like a team. One month we might need to increase two or three units [of insulin]. The following month we might decrease it or increase it. The relationship between physician and patient needs to be good. So you all feel like you both care. Not only the physician, but also the patient, it is take and give. [Group 2: Patient 6]* *They have the belief that, with doctors, all the times they go there, they [physicians] make him sick; not by medications but by giving a new diagnosis. [Group 2: Provider 4]* *My doctor is the same [referring to another participant’s comment] if I don’t get sick, and I don’t visit him and ask him for blood sugar test every 3 months, he won’t do it. My doctor never calls me or asks me to come do lab tests for my blood sugar, unless I go by myself…I believe it was a cultural barrier, I think they just write prescription and that is it. The next time I went to the doctor, they do not ask me about the results or any of that. You know, they should ask. They don’t. [Group 3: Patient 4]*

## Discussion

To our knowledge, this is the first study exploring the cultural influences on DSM from the perspective of Arab American patients with diabetes and Arab American providers. While we are unable to generalize our findings beyond our small sample, the themes presented here suggest several potential avenues for future inquiry into DSM and associated outcomes among Arab American with diabetes. One challenge provider participants identified was the lack of basic educational materials available to Arab American patients with diabetes. Arabic language resources are becoming increasingly available (see: http://patienteducation.stanford.edu/programs/cdsmp.html). Nonetheless, findings suggest that at least some DSME materials could be further improved by being tailored to Arab cultural norms and traditions that impact DSM, such as specific DSM instructions during the holy month of Ramadan.

Another area of concern was the lack of Arabic speaking multidisciplinary teams available to assist Arabic speaking patients. In the absence of sufficient bilingual allied health staff, this barrier may be most feasibly overcome through the use of over-the phone interpreter services. While these devices are now commonplace in many hospital settings, their use in primary care and related outpatient settings is unclear. Nonetheless, evidence suggests that using such devices may be cost effective and ultimately can contribute to improved patient care in low English language proficiency populations [22, 23]. Another way to augment the DSM counseling provided by the physician is the use of group format DSM interventions. While purely speculative at this time, group-based interventions may also serve to reduce stigma and increase social supports for those facing DSM. One example of this approach is the Stanford diabetes self-management program, which has been shown to improve self-management practices across a range of conditions and ethnic groups. While the Stanford diabetes specific program is not available in Arabic, future research could address programs for use among Arab American populations.

An equally important finding was the different perspectives on the patient-provider relationship. Although both groups acknowledged patients’ dissatisfaction with clinical encounters, reasons for dissatisfaction differed. If providers believed that patient satisfaction/dissatisfaction stems from cultural beliefs alone, then it was unlikely that they modified other care processes that may have contributed to patient dissatisfaction. Further, the diversity of perspectives arising from this small sample suggests further research is warranted to better identify the root causes of patient dissatisfaction. A better understanding of these dynamics is necessary for improving patient provider interactions among this population.

Similarly, findings also suggest disparate views regarding the family’s role in supporting DSM. Providers actively recruited family members as partners in supporting DSM. While providers felt that this was an effective strategy, considering the gaps in DSM education at the individual level, it is plausible that family members are equally ill informed about DSM. Moreover, while our study did not explicitly query this domain, patients’ comments about stress and recent research in the area of family involvement in DSM suggests that family members may be unprepared for this role and that patients and family members may experience emotional distress as a result [24]. Family-centered interventions are gaining in popularity [25, 26]. Given the family’s central role in the Arab culture, future research is warranted to examine whether or not intervening at the family level enhances the effectiveness of future DSM interventions in the Arab American population.

## Limitations

Despite the important contributions of this current work, study findings are limited to the small convenience sample of Arab American patients and providers recruited from one geographic area. This sample also includes a high percentage of recently emigrated Arab Americans immigrants who had limited formal education. These findings should therefore be interpreted with caution and may not reflect the challenges faced by other healthcare providers who care for Arab American patients or the opinions of other Arab Americans with diabetes.

## Conclusions

Study findings suggest several domains that should be considered in future work and that hold relevance for clinical practice. Specifically, the current study suggests that there is a need to develop linguistically and culturally reliant DSM educational materials and relevant supports for use in the Arab American population. Further, the findings draw attention to the differing views that providers and patients have on the role of family members in supporting DSM efforts and why some patients feel dissatisfied with clinical encounters. Informed by these findings, we are currently working to develop a family-centered DSM intervention and systematically evaluate the effects of this approach on DSM outcomes in Arab Americans with diabetes.

## References

[1] Agency for Healthcare Research and Quality. Patient self-management support programs: An evaluation. In. Rockville; 2014. Retrieved from http://www.ahrq.gov/research/findings/final-reports/ptmgmt/index.html.

[2] Nam S, Chesla C, Stotts NA, Kroon L, Janson SL (2011). Barriers to diabetes management: patient and provider factors. Diabetes Res Clin Pract.

[3] Shortus T, Kemp L, McKenzie S, Harris M (2013). ‘Managing patient involvement’: provider perspectives on diabetes decision-making. Health Expect.

[4] Aboul-Enein BH, Aboul-Enein FH (2010). The cultural gap delivering health care services to Arab American populations in the United States. J Cult Divers.

[5] Berlie HD, Herman WH, Brown MB, Hammad A, Jaber LA (2008). Quality of diabetes care in Arab Americans. Diabetes Res Clin Pract.

[6] Hammad A, Rashid K, Rabah R, Hassoun R, Connelly M. Guide to Arab culture: Health care delivery to the Arab American community. Dearborn, MI: Arab Community Center for Economic and Social Services (ACCESS); 1999.

[7] Arab American Institute. Demographics; 2015. Retrieved from www.aaiusa.org/demographics.

[8] Aswad M. Health survey of the Arab, Muslim, and Chaldean American communities in Michigan. Dearborn, MI: Arab Community Center for Economic and Social Services (ACCESS); 2001.

[9] Gennesee County Health Department. Arab American health survey 2003. In. 2003.

[10] Hassoun R, Nassar-McMillan SS (2013). A bioanthropological perspective of hypertension in Arab Americans in the metropolitan area. Biopsychosocial perspectives on Arab Americans : culture, development, and health.

[11] Jaber LA, Slaughter RL, Grunberger G (1995). Diabetes and related metabolic risk factors among Arab Americans. Ann Pharmacother.

[12] Dallo FJ, Borrell LN (2006). Self-reported diabetes and hypertension among Arab Americans in the United States. Ethn Dis.

[13] Jaber LA, Brown MB, Hammad A, Nowak SN, Zhu Q, Ghafoor A, Herman WH (2003). Epidemiology of diabetes among Arab Americans. Diabetes Care.

[14] Jadalla A, Lee J (2012). The relationship between acculturation and general health of Arab Americans. J Transcult Nurs.

[15] Jamil H, Fakhouri M, Dallo F, Templin T, Khoury R, Fakhouri H (2008). Disparities in self-reported diabetes mellitus among Arab, Chaldean, and black Americans in Southeast Michigan. J Immigr Minor Health.

[16] Kridli SA, Herman WH, Brown MB, Fakhouri H, Jaber LA (2006). The epidemiology of diabetes and its risk factors among Chaldean Americans. Ethn Dis.

[17] Carbone ET, Rosal MC, Torres MI, Goins KV, Bermudez OI (2007). Diabetes self-management: perspectives of Latino patients and their health care providers. Patient Educ Couns.

[18] Chin MH, Cook S, Jin L, Drum ML, Harrison JF, Koppert J, Thiel F, Harrand AG, Schaefer CT, Takashima HT, Chiu SC (2001). Barriers to providing diabetes care in community health centers. Diabetes Care.

[19] Boyatzis RE (1998). Transforming qualitative information : thematic analysis and code development.

[20] Holloway I, Wheeler S, Holloway I (2010). Qualitative research in nursing and healthcare.

[21] Hsieh HF, Shannon SE (2005). Three approaches to qualitative content analysis. Qual Health Res.

[22] Guba EG, Lincoln YS (1989). Fourth generation evaluation.

[23] Tong A, Sainsbury P, Craig J (2007). Consolidated criteria for reporting qualitative research (COREQ): a 32-item checklist for interviews and focus groups. Int J Qual Health Care.

[24] Jacobs EA, Shepard DS, Suaya JA, Stone EL (2004). Overcoming language barriers in health care: costs and benefits of interpreter services. Am J Public Health.

[25] Pottie K, Batista R, Mayhew M, Mota L, Grant K (2014). Improving delivery of primary care for vulnerable migrants: Delphi consensus to prioritize innovative practice strategies. Can Fam Physician.

[26] Sperry S, Knox B, Edwards D, Friedman A, Rodriguez M, Kaly P, Albers M, Shaffer-Hudkins E (2014). Cultivating healthy eating, exercise, and relaxation (CHEER): A case study of a family-centered and mindfulness-based cognitive-behavioral intervention for obese adolescents at risk for diabetes and cardiovascular disease. Clin Case Stud.

