# Erratum to: Diabetes self-management among Arab Americans: patient and provider perspectives

**DOI:** 10.1186/s12914-016-0098-7

**Published:** 2016-09-20

**Authors:** Heather Fritz, Rosanne DiZazzo-Miller, Elizabeth A. Bertran, Fredrick D. Pociask, Sandra Tarakji, Judith Arnetz, Catherine L. Lysack, Linda A. Jaber

**Affiliations:** 1 Institute of Gerontology, Wayne State University, 87 East Ferry Street, 226 Knapp Building, Detroit, MI 48202 USA; 2 Department of Health Care Sciences, Wayne State University, 259 Mack Ave, Detroit, MI 48201 USA; 3 Department of Pharamacy Practice, Wayne State University, 259 Mack Ave, Detroit, MI 48201 USA; 4 Department of Family Medicine and Public Health Sciences, Wayne State University, Detroit, MI 48202 USA; 5 Department of Family Medicine, Michigan State University, 788 Service Rd., B103 Clinical Center, East Lansing, MI 48824 USA

Unfortunately, the original version of this article [1] contained an error. The acknowledgements section was included incorrectly and should have acknowledged The Martha Schnebly Endowed Research Fund.
